# Emerging roles of ATG9/ATG9A in autophagy: implications for cell and neurobiology

**DOI:** 10.1080/15548627.2024.2384349

**Published:** 2024-08-04

**Authors:** Jiyoung Choi, Haeun Jang, Zhao Xuan, Daehun Park

**Affiliations:** a Department of Medical and Biological Sciences, The Catholic University of Korea, Bucheon, South Korea; b Department of Biotechnology, The Catholic University of Korea, Bucheon, South Korea; c School of Biology and Ecology, University of Maine, Orono, ME, USA

**Keywords:** ATG proteins, ATG9, ATG9A, autophagy, lipid scramblase, phagophore expansion

## Abstract

Atg9, the only transmembrane protein among many autophagy-related proteins, was first identified in the year 2000 in yeast. Two homologs of Atg9, ATG9A and ATG9B, have been found in mammals. While ATG9B shows a tissue-specific expression pattern, such as in the placenta and pituitary gland, ATG9A is ubiquitously expressed. Additionally, ATG9A deficiency leads to severe defects not only at the molecular and cellular levels but also at the organismal level, suggesting key and fundamental roles for ATG9A. The subcellular localization of ATG9A on small vesicles and its functional relevance to autophagy have suggested a potential role for ATG9A in the lipid supply during autophagosome biogenesis. Nevertheless, the precise role of ATG9A in the autophagic process has remained a long-standing mystery, especially in neurons. Recent findings, however, including structural, proteomic, and biochemical analyses, have provided new insights into its function in the expansion of the phagophore membrane. In this review, we aim to understand various aspects of ATG9 (in invertebrates and plants)/ATG9A (in mammals), including its localization, trafficking, and other functions, in nonneuronal cells and neurons by comparing recent discoveries related to ATG9/ATG9A and proposing directions for future research.

**Abbreviation**: AP-4: adaptor protein complex 4; ATG: autophagy related; cKO: conditional knockout; CLA-1: CLArinet (functional homolog of cytomatrix at the active zone proteins piccolo and fife); cryo-EM: cryogenic electron microscopy; ER: endoplasmic reticulum; KO: knockout; PAS: phagophore assembly site; PtdIns3K: class III phosphatidylinositol 3-kinase; PtdIns3P: phosphatidylinositol-3-phosphate; RB1CC1/FIP200: RB1 inducible coiled-coil 1; SV: synaptic vesicle; TGN: trans-Golgi network; ULK: unc-51 like autophagy activating kinase; WIPI2: WD repeat domain, phosphoinositide interacting 2.

## Introduction

Macroautophagy/autophagy is a crucial self-degradation mechanism in eukaryotic cells [1–3]. It is triggered by various stresses, such as starvation, inflammation, and ischemia, and plays a fundamental role in preserving cellular homeostasis [3–6]. Upon initiation, autophagy involves the encapsulation of aged cellular constituents, including organelles and the cytoplasm, within double-membrane structures known as autophagosomes, which then undergo subsequent fusion with lysosomes, leading to the formation of autolysosomes [1,7,8]. This fusion results in the degradation of both the lumenal contents and the inner membrane of the autophagosome through the enzymatic action of lysosomal hydrolases [1,7,8].

Numerous *ATG* (autophagy related) genes have been identified in the yeast *Saccharomyces cerevisiae*, leading to the subsequent identification of homologs in mammals [9,10]. To date, more than 40 ATG proteins have been recognized as essential for autophagy [9]. They are typically assembled into multi-subunit complexes and work together to orchestrate autophagosome biogenesis [9,10]. In mammals, core ATG proteins can be categorized into five different groups: (i) the ULK (unc-51 like autophagy activating kinase) complex, (ii) the class III phosphatidylinositol 3-kinase (PtdIns3K) complex, (iii) the ATG12 conjugation machinery, (iv) the Atg8/LC3 conjugation machinery, and (v) the ATG9A vesicles and their interactors, such as ATG2A [2,11–13].

**Figure 1.**
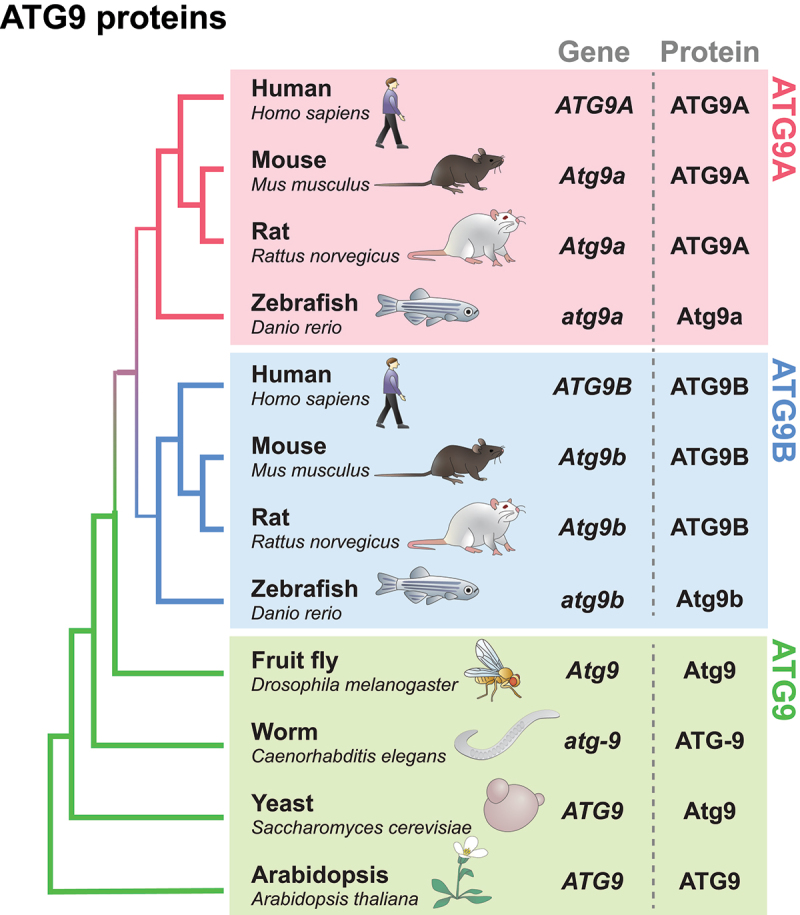
Cladogram and nomenclature of ATG9 proteins. The phylogenetic cladogram from a multiple sequence alignment of ATG9 proteins across different species. The amino acid sequences (NCBI) from each species were aligned using the alignment software MUSCLE [17], and the phylogenetic tree was created using the maximum likelihood method in MegaX [18] and then verified with panther tree viewer on ALLIANCE genome resources [19] and the ensembl gene tree [20]. The gene and protein nomenclature of each homolog are indicated on the right side. Notably, two homologs (A/B) are expressed in mammals and zebrafish, while only one homolog exists in flies, worms, yeast, and *Arabidopsis*.

Among the various types of core autophagy proteins, ATG9 is the only integral membrane protein expressed ubiquitously and is thought to localize to small vesicles [14–16]. However, the specific role of ATG9 in the autophagic process and the precise stage in which it is involved have long remained unknown. In less complex organisms such as invertebrates, only one type of ATG9 (Figure 1) is present and is thought to play a pivotal role in autophagosome biogenesis [16,21]. However, in most vertebrates, including mammals, two homologs of ATG9 are expressed: ATG9A and ATG9B (Figure 1) [21,22]. The nomenclature of each homolog in each model organism is shown in Figure 1.

Two homologs, ATG9A and ATG9B, have similar sizes and topologies. In addition, they are thought to share common functions in autophagy [21,23]. However, ATG9A and ATG9B clearly show different tissue expression patterns [21]. Like ATG9 in invertebrates, ATG9A is expressed ubiquitously across various tissues, with high expression in the brain and spinal cord [24,25], while ATG9B exhibits a very restricted distribution and is exclusively expressed in the placenta and pituitary gland, with low expression in the testis and uterus [25]. Whether ATG9B merely provides functional redundancy [21,26] or possesses distinct functions in specific cell types [27] remains unknown. However, recent studies suggest the interesting possibility that ATG9B may have acquired novel functions during placental evolution, while ATG9A is believed to retain more conserved functions of the parental gene [21]. Therefore, ATG9B may play a primary role, especially in placental development, but it may also perform functions similar to those of ATG9A in certain situations, based on its sequence and structural similarities.

However, ATG9A undoubtedly plays a major role in autophagy in most tissues due to its evolutionary similarity to ancestral functions [21], wide tissue expression patterns [24,25,28], and striking knockout (KO) phenotypes, including autophagy defects [29–36] (see sections 5 and 7). Furthermore, high levels of ATG9A in the central nervous system underscore its vital role in neurons, where the regulation of autophagy for dysfunctional organelles and damaged proteins is crucial due to their elevated metabolic activity in response to continuous stimuli [37–39]. Therefore, in this review, we will focus on recent findings on ATG9 (in invertebrates and plants) and ATG9A (in mammals) (Figure 1).

## The structure of ATG9/ATG9A

### Transmembrane topology of ATG9/ATG9A

**Figure 2.**
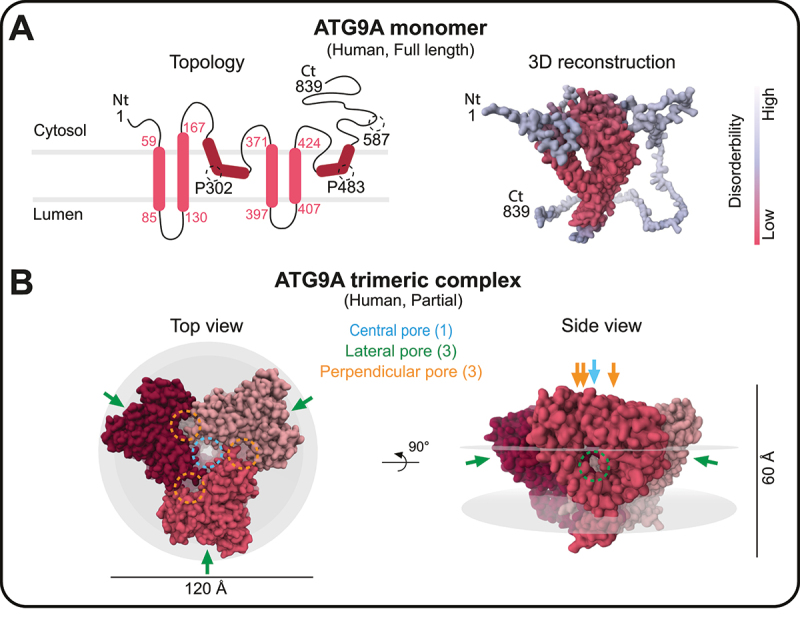
The structure of the human ATG9A. (A) left panel: the topology of the human ATG9A monomer (full length; 1–839 amino acids). The numbers in red indicate the corresponding amino acid residues of each alpha helix. Dark red regions show the two membrane-embedded helices that do not penetrate the membrane. Right panel: the 3D reconstruction from the AlphaFold prediction [43] of human ATG9A (AF-Q7Z3C6-F1-model_v1). The structure is color-coded by the disordered score. (B) the structure of the human ATG9A trimer generated from the cryo-EM density map (EMDB: EMD-21876, PDB: 6WR4). Each monomer is colored differently, and gray disks outline the edges of the membrane. Note that this model does not represent the full-length protein, and shows residues 36 to 587, with missing loop residues 96–108 and 536–538, and includes two additional helices in the C-terminal domain among a total of 839 amino acids in a monomer. The arrows show the locations of the three different types of pores. The structural images were generated and modified using the RCSB PDB webserver (https://www.rcsb.org) [44] and 3D viewers Mol* viewer [45], respectively.

The ATG9/ATG9A protein varies in length among different species, typically ranging from 700 to 1000 amino acids [21]. The central region is consistently observed across all species, encompassing approximately 500 amino acids and featuring six major alpha helices [21]. All six helices were previously considered transmembrane regions based on sequence analysis [28], which seems to be supported by a cryogenic electron microscopy (cryo-EM) analysis of ATG9 from *Arabidopsis thaliana* [40]. However, recent studies using higher-resolution cryo-EM have revealed that ATG9A in humans, Atg9 in yeast, and ATG9 in *Arabidopsis* have four transmembrane regions that are highly conserved across species [33,41,42]. The misconception of the initial ATG9 structure was due to the presence of two alpha helices in ATG9/ATG9A that do not penetrate the membrane but are buried in the cytosolic leaflet due to the presence of a proline located in the middle of each alpha helix (P302 and P483), which bends the entire configuration of the helices (Figure 2A; left panel) [41,42]. Both the N- and C-termini of ATG9/ATG9A, which exhibit varying lengths across species, consistently reside in the cytosol (Figure 2A; left panel) [28] and are highly disordered (Figure 2A; right panel) [42]. This characteristic may support a functional role for this region, enabling weak-multivalent interactions that underlie the liquid‒liquid phase separation of ATG9A vesicles from other proteins [46].

### The trimeric architecture of ATG9/ATG9A

The initial cryo-EM study revealed that ATG9 forms a homotrimer in *Arabidopsis* [40]. Consistently, subsequent high-resolution cryo-EM studies in yeast (Atg9) and humans (ATG9A) showed that the trimer adopts a triangular configuration through domain-swapped interactions (Figure 2B) [33,41,42]. Notably, the trimer forms three distinctive types of pores: a central pore (or ventral pore), located in the core of the structure and penetrating the membrane vertically (blue in Figure 2B) [33,41,42]; three lateral pores, oriented parallel to the membrane and forming tunnels that connect to the external environment (green in Figure 2B) [33,41,42]; and three perpendicular pores, which are suggested to create extra vertical cavities that could connect the central pore and lateral pores (orange in Figure 2B) [33]. This intricate cavity structure of ATG9/ATG9A creates a funnel-shaped solvent pool in the membrane, facilitating the movement of phospholipids between the two lipid leaflets in the double membrane, which suggests that ATG9/ATG9A may function as a lipid scramblase [41,42,47] (see section 6).

Furthermore, ATG9/ATG9A can undergo a conformational change that significantly influences the diameter of the central pore; the upper part becomes larger, and the bottom part becomes smaller [41,42]. Similar structural changes are observed in ABC (ATP-binding cassette) transporters, transmembrane proteins that facilitate substance translocation across the membrane, and may provide insights into the function of ATG9/ATG9A as a lipid scramblase [41] (see section 6). Another study speculated that the entrance of the central pore is too small to permit only water molecules, which are much smaller than the phospholipids [33]. In this case, the central pore might function as a water channel, regulating the osmotic pressure of the vesicle [33]. The results of molecular dynamics simulations further indicate that conformational changes in ATG9/ATG9A may cause membrane bending [33,42] and facilitate the preferential positioning of the molecule in highly curved regions [48]. Additionally, a hexameric form of yeast Atg9, characterized by a head-to-head association between two distinct trimers localized on two different membranes, has been observed in vitro [42]. However, whether these interactions indeed occur in vivo, particularly in human ATG9A trimers, remains unclear. Nevertheless, the results provide us with an intriguing concept regarding the contact site. The interaction between two trimers of ATG9/ATG9A localized on different membranes may generate forces to establish a contact site between the freely flowing ATG9/ATG9A vesicles and other organelles, such as the phagophore, and this interaction might be crucial for phagophore expansion and growth.

## Subcellular trafficking of ATG9/ATG9A

### Trafficking in nonneuronal cells

**Figure 3.**
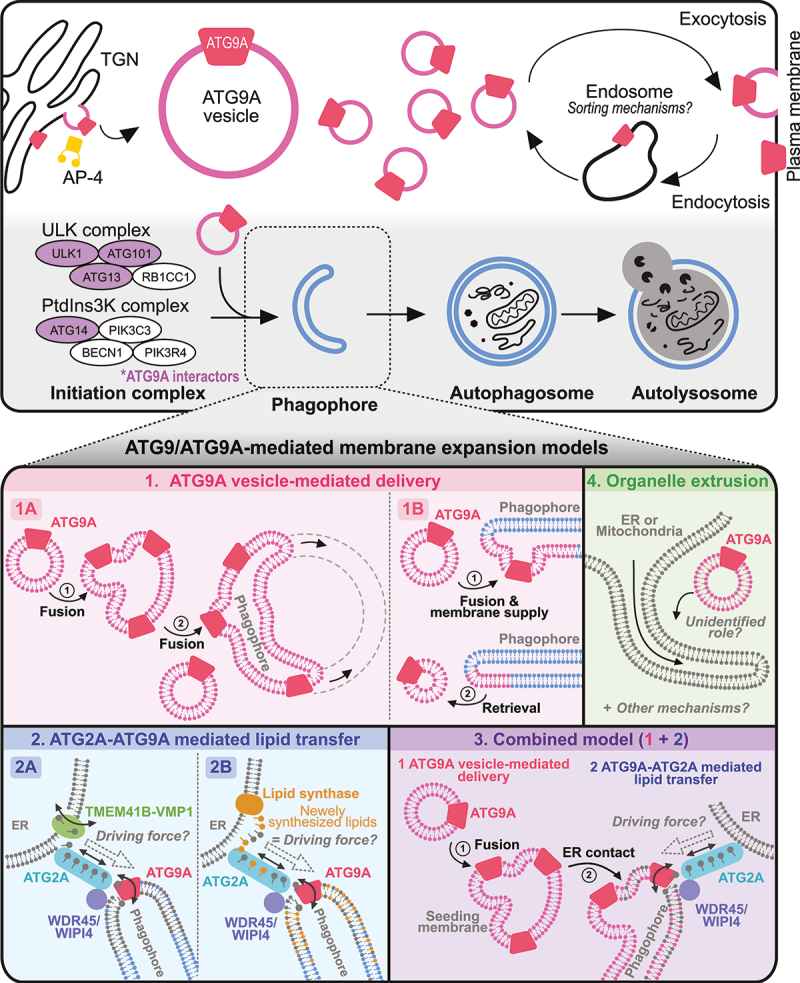
ATG9/ATG9A trafficking in nonneuronal cells and working models for phagophore growth. Upper panel: trafficking of ATG9/ATG9A in nonneuronal cells and its involvement in the autophagic process. Proteins shown in purple are potential ATG9/ATG9A interactors according to previous proteomic studies. Lower panel: proposed models for ATG9/ATG9A-mediated phagophore growth and the remaining questions.

In mammalian cells, ATG9A is widely distributed across various subcellular organelles, such as the trans-Golgi network (TGN), endosomal compartments, and plasma membrane (Figure 3; upper panel) [28,31,46,49–53]. However, the exact trafficking mechanisms of ATG9A remain to be determined. One well-defined interactor of ATG9A, known to regulate its trafficking in cells, is AP-4 (adaptor protein complex 4) [31,54–57]. Independent proteomic studies have reported AP-4 subunits as top hits for ATG9A [46,58,59]. In addition, ATG9A has a conserved YXXΦE motif in its cytosolic N-terminal region, which strongly binds to the μ subunit of AP-4, leading to the budding of vesicles from the TGN into the peripheral compartments (Figure 3; upper panel) [31]. Indeed, the inhibition of ATG9A-AP-4 interactions by the KO of the *AP4E1/AP-4 ε* (adaptor related protein complex 4 subunit epsilon) gene or mutations in the YXXΦE motif of ATG9A results in the massive accumulation of ATG9A at the TGN [31]. Other adaptor protein complexes, such as AP-1 and AP-2, are also known to bind to ATG9A [51,60] and regulate its trafficking [52,61,62], but their binding is much weaker than that of AP-4 [31]. These results suggest that AP-4 plays a primary role in ATG9A trafficking but that other adaptor proteins are also likely required in different subcellular compartments.

### Trafficking in neurons

**Figure 4.**
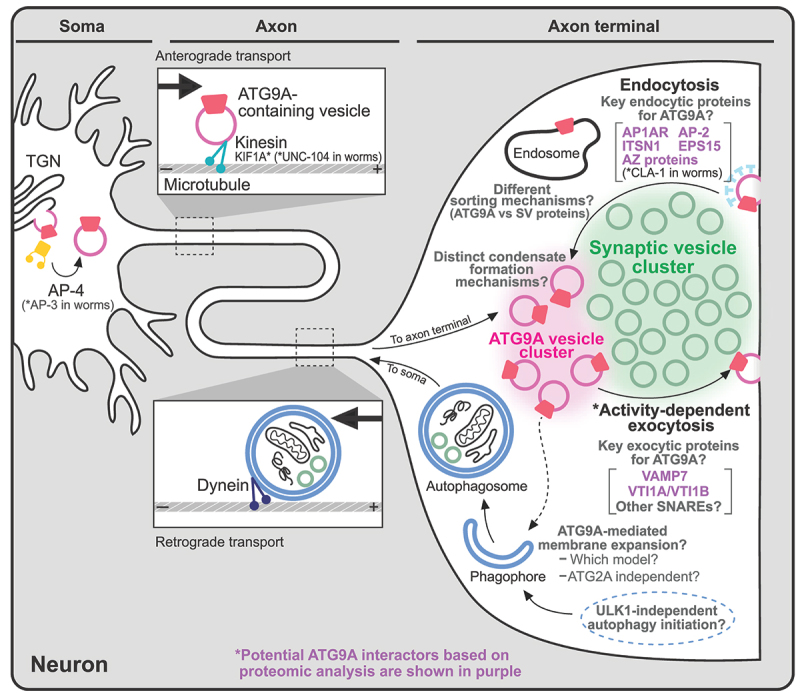
Trafficking and function of ATG9/ATG9A in neurons. ATG9/ATG9A is believed to be transported to nerve terminals, undergo activity-dependent exo- and endocytosis, and localize to distinct vesicle pools at presynapses. However, the details of its precise roles in nerve terminals remain unclear. Potential ATG9A interactors based on proteomic analyses are shown in purple. The asterisk (*) indicates evidence from worms (*C. elegans*).

In neurons, similar to nonneuronal cells, ATG9A is localized in the TGN, and AP-4 is crucial for its exit from the TGN [56]. Interestingly, in *Caenorhabditis elegans*, which do not express AP-4, AP-3 serves as a functional substitute for mammalian AP-4 (Figure 4) [63]. In neurons, ATG9/ATG9A is also enriched in nerve terminals; thus, vesicles containing ATG9/ATG9A generated in the soma need to be transported to the presynapse, where synaptic transmission occurs to relay information to the next neuron [56,63–65]. Indeed, in *C. elegans* neurons, ATG-9-containing vesicles undergo anterograde transport along the axon by KIF1A (kinesin family member 1A)/UNC-104, a member of the kinesin superfamily responsible for transporting synaptic vesicles (SVs) (Figure 4) [64], and the ATG-9 vesicles undergo exo-/endocytosis at the presynapse in response to neuronal stimulation (Figure 4) [63]. The endocytic process is mediated by well-defined endocytic proteins such as AP-1, AP-2, EPS15 (epidermal growth factor receptor pathway substrate 15) and ITSN1 (intersectin 1) [65]. These endocytic proteins are also expressed in mammalian presynapses, suggesting the existence of a conserved ATG9/ATG9A trafficking pathway in nerve terminals across different species. In addition, evidence shows that the long isoform of CLA-1 (CLArinet, functional homolog of cytomatrix at the active zone proteins piccolo and fife), an active zone protein in *C. elegans* with functional similarity to vertebrate piccolo and bassoon, is involved in the presynaptic sorting of ATG-9 (Figure 4) [65,66]. In addition, recent studies have provided either direct or indirect evidence showing the importance of endocytic and active zone proteins for ATG9A trafficking or presynaptic autophagy at mammalian presynapses. For instance, loss-of-function mutations in endocytic proteins in neurons, such as *Dnm1* (dynamin 1) and *Dnm3* double KO neurons or *synaptojanin1* KO neurons, lead to massive accumulation of ATG9A in presynaptic nerve terminals [63]. These results suggest that endocytic proteins play a crucial role in normal ATG9A trafficking in mammalian synapses. Additionally, a study reported that bassoon is a key regulator of presynaptic autophagy in mammalian synapses [67]. However, since this process is thought to be mediated mainly by interactions between ATG5 and bassoon, future investigations are essential to explore the involvement of active zone proteins in mammalian presynaptic ATG9A trafficking.

The presynaptic localization of ATG9/ATG9A [56,63–65] and its SV-like behaviors, such as activity-dependent exo-/endocytosis [63], have raised questions about its precise localization in nerve terminals: does it localize to SVs or a distinct vesicle population? Two recent studies [46,59] shed light on this matter, and both suggest that ATG9A localizes to distinct vesicles rather than to SVs (Figure 4). First, using an ectopic expression system, we previously reported that ATG9A and synaptophysin, a major integral membrane protein of SVs, form two distinct vesicle clusters in nonneuronal cells when co-expressed with synapsin, a peripheral SV protein [46]. A proteomic analysis of these vesicles extracted from nonneuronal cells further revealed a different protein composition [46]. Second, using a proteomic analysis and super-resolution microscopy, Binotti et al. reported that ATG9A vesicles represent a distinct vesicle population in nerve terminals [59]. Furthermore, in *C. elegans* studies, the abnormal accumulation of ATG-9 at presynapses in *cla-1* mutants was not observed with other SV proteins (e.g., the integral SV membrane protein synaptogyrin and membrane-bound GTPase activating protein Rab3), suggesting that a distinct sorting mechanism for ATG9/ATG9A vesicles exists (Figure 4) [65].

However, an inconsistency regarding the size of ATG9A vesicles relative to SVs has been noted between the two studies [46,59]. We previously reported that ATG9A vesicles (55 nm) were slightly larger than synaptophysin vesicles (42 nm) when measured within the same cell using an ectopic expression system (COS7 cells) and assessed via correlative light and electron microscopy (CLEM) [46]. However, a recent study revealed that ATG9A vesicles are similar in size to SVs when immune-isolated from the rat brain or imaged in cultured hippocampal neurons using super-resolution microscopy [59]. These results may suggest variations in the size of ATG9A vesicles between neurons and nonneuronal cells, or overexpressing ATG9A may alter the vesicle size due to its intrinsic ability to induce membrane bending [33,42]. We cannot exclude the possibility that different procedures, such as fixation or dehydration steps for sample preparation, might alter the size of vesicles [68,69]. Consequently, more evidence for the morphology and function of ATG9A vesicles in nerve terminals is needed.

## The properties of ATG9/ATG9A-containing vesicles

Although previous findings indicate that ATG9A localizes to vesicles at presynaptic terminals, which are segregated from SV clusters [46,59], the mechanism of vesicle segregation is unclear. The differences in size, composition of proteins and lipids, and affinity for synapsin might provide forces for the separation of the two different types of vesicles.

However, the distinct localization of ATG9A in nerve terminals led us to compare recent proteomic analyses of ATG9A performed by us and other groups to identify common ATG9A interactors. Thus, we compared three independent proteomic studies [46,58,59] that analyzed ATG9A from different sources using different techniques. Kannangara et al. used a proximity biotinylation technique, BioID, to investigate ATG9A interactions in HEK293T cells (exogenous) [58]. We previously performed immunoisolation of ATG9A vesicles from COS7 cells expressing ATG9A-HA (exogenous) and conducted TurboID-based proximity biotinylation of COS7 cells expressing ATG9A-miniTurboID-HA (exogenous) [46]. Binotti et al. purified presynaptic ATG9A vesicles from rat brain synaptosomes (endogenous) and determined the proteome of the immuno-isolated ATG9A-containing vesicles using label-free quantitative mass spectrometry [59].

The proteins commonly identified across all three independent studies are very limited, which might be due to the different experimental conditions for the analyses. However, some endocytic proteins, AP1AR (adaptor related protein complex 1 associated regulatory protein), ITSN1, and EPS15, were enriched in all three datasets [46,58,59]. Other adaptor protein subunits, such as AP-3 and AP-4, were abundant in nonneuronal ATG9A vesicles [46,58], but only AP-2 subunits were identified from ATG9A vesicles in neuronal synaptosomes [59], supporting the involvement of different adaptor proteins according to the different subcellular localizations of ATG9A (note that proteins enriched in the TGN, such as AP-4, are excluded from synaptosomal fractions) (Figure 4).

Recycling of ATG9A vesicles has been suggested for both nonneuronal and neuronal cells [61,63,70], but the specific SNARE proteins responsible for the fusion of ATG9A vesicles remain elusive. A proteomic analysis may shed light on this matter. For example, VTI1B (vesicle transport through interaction with t-SNAREs 1B), a SNARE protein, was consistently identified in all three studies [46,58,59]. VAMP7 was identified from two independent studies [46,59]. VTI1A, another SNARE protein, was found only in the ATG9A vesicles in nerve terminals (Figure 4) [59]. These SNAREs might define the functional distinction of ATG9A-containing vesicles.

Notably, all three results showed that ATG9A vesicles generally lack proteins that are enriched in SVs [46,58,59], suggesting that ATG9A vesicles and SVs follow different trafficking pathways. Furthermore, ULK1, ATG13 and ATG101, components of the ULK complex, and ATG14 in the PtdIns3K complex, which are required for autophagy initiation steps [2,11–13], were particularly abundant in nonneuronal ATG9A vesicles (Figure 2) [46,58] but not in synaptosomes [59,71] (see the next section), suggesting that neurons may use a different machinery to initiate the formation of autophagosomes in nerve terminals (Figure 4).

## Role of ATG9/ATG9A in autophagy

Autophagy is a complicated signaling pathway that contributes to cellular metabolism and cell survival [1–3]. Under basal conditions, the activity of the MTOR (mechanistic target of rapamycin kinase) complex suppresses autophagy induction, but autophagy is triggered by various conditions, such as starvation, inflammation, and ischemia, through the downregulation of the MTOR complex [5,6]. Given that ATG9/ATG9A interacts with molecules involved in autophagy initiation [72–74], it may play a specific role in the very early stages of the autophagic process, particularly during the formation of phagophore assembly sites (PASs).

The ULK complex, which includes the kinases ULK1/ULK2, ATG13, ATG101, and RB1CC1/FIP200 (RB1 inducible coiled-coil 1), is regarded as the primary regulator of autophagosome biogenesis [2,11–13]. ATG9A vesicles are thought to translocate to autophagy initiation sites in a ULK1-dependent manner [28,50,60], and proteomic analyses further identified ULK1, ATG13, and ATG101 as potential molecules interacting with ATG9A (Figure 3; upper panel) [46,58,59]. A recent structural study revealed that the cytosolically extended C-terminal tail of ATG9A interacts with a cleft at the ATG13-ATG101 interface during mitochondrial damage-induced autophagy (mitophagy) [72]. In addition, structural changes in ATG13 and ATG101 may facilitate their interaction with ATG9A, guiding them to the initiation complex at the PAS [73].

The PtdIns3K complex, which consists of PIK3C3/VPS34 (phosphatidylinositol 3-kinase catalytic subunit type 3), PIK3R4/VPS15/p150 (phosphoinositide-3-kinase regulatory subunit 4), BECN1 (beclin 1), ATG14, and NRBF2 (nuclear receptor binding factor 2), is activated by phosphorylation by the ULK1 complex [2,11–13]. This complex generates phosphatidylinositol 3-phosphate (PtdIns3P), which facilitates the recruitment of specific PtdIns3P effector proteins, such as those in the WIPI1, WIPI2, WDR45B/WIPI3, WDR45/WIPI4 family [11–13]. PtdIns3P serves as a docking site that recruits WDR45/WIPI4 (WD repeat domain 45), thereby facilitating the localization of its binding partner, ATG2A, to growing phagophore sites [11–13]. The interaction between ATG2A and ATG9A, which involves lipid transfer and equilibration, is thought to be essential for the expansion of the phagophore membrane [47,75] (see the next section). Moreover, PIK3C2A (phosphatidylinositol-4-phosphate 3-kinase catalytic subunit type 2 alpha) also produces PtdIns3P, which has been found to interact with ATG9A and ATG14 [74]; this finding is further supported by proteomic analyses [46,58].

After the initiation complex grows to the phagophore, ATG9/ATG9A is believed to interact dynamically with phagophores and autophagosomes without being incorporated into them [50]. Furthermore, ATG9A is present in limited amounts on the phagophore [35,50], suggesting that a small amount of ATG9A is sufficient for membrane expansion. Various working models exist to describe how ATG9A may be involved as the membrane of the phagophore expands to complete autophagosome formation. Here, we summarize these models as follows: (1) ATG9A vesicle-mediated delivery, (2) ATG2A-ATG9A-mediated lipid transfer, (3) a combined model, or (4) extrusion from other organelles (Figure 3) (see the next section).

Although the exact mechanisms by which ATG9A is involved in autophagosomal membrane expansion remain elusive, the absence of ATG9A results in marked defects in autophagy in various organisms [29–36]. In ATG9A-deficient cells, abnormal accumulation of the ULK1 complex and SQSTM1/p62 (sequestosome 1), a classical selective autophagy receptor, at the site of autophagosome formation was observed [32]. However, the level of LC3B, a marker of autophagy, in ATG9A-deficient cells has been shown to vary under different experimental conditions [29,33–35,47]. For example, some previous studies reported large and bright LC3B puncta corresponding to aberrant autophagosomes or protein aggregates in *ATG9A* KO HeLa cells under resting conditions [33,34]. However, during starvation or bafilomycin A_1_ treatment, LC3B fails to be recruited to autophagosomes in *Atg9a* KO MEFs [29] and *ATG9A* KO HEK293 cells [35,47]. Despite the complex behaviors of LC3B, all the results indicate that the deletion of *Atg9a/ATG9A* impairs the normal growth of the phagophore and protein degradation, leading to the accumulation of various initiation factors. Notably, the expression of ATG9B can rescue the abnormal accumulation of SQSTM1 in *ATG9A* KO cells [21], suggesting a potential overlapping function between the two proteins, as we discussed earlier. However, this rescue may not occur in all cells in vivo due to the restricted tissue distribution pattern of ATG9B [21,22].

## ATG9/ATG9A-mediated membrane expansion models

The structure, localization, and KO phenotypes strongly indicate the involvement of the ATG9A protein in the autophagic process, especially in the initiation steps. However, researchers have not clearly determined how ATG9A can affect the initiation and growth of phagophores [11]. Therefore, in this section, we aim to examine the proposed working models of ATG9/ATG9A in phagophore growth (Figure 3; lower panels; Models 1 to 4).

### ATG9/ATG9A vesicle-mediated delivery (Figure 3; models 1A and 1B)

During autophagy, a substantial amount of membrane is added to the growing phagophore [11]. Considering the localization of ATG9/ATG9A on small vesicles as a transmembrane protein and its interactions with other core autophagy proteins, ATG9A vesicles appear to be a suitable membrane source for phagophore expansion through direct fusion events [76,77]. This concept is simple and intuitive but may not be supported by experimental data showing the localization of ATG9/ATG9A. According to this idea, ATG9/ATG9A vesicles continue to fuse to expand a phagophore; thus, a significant amount of ATG9/ATG9A should be present in the growing phagophore (Figure 3; Model 1A) [14]. However, ATG9A was not found on the autophagosome membrane in HEK293 cells [50]. In addition, ATG9A was not identified in a proteomic analysis of autophagosomes in mouse neurons [78]. Other studies have also reported the absence or presence of a very low amount of ATG9A in autophagosomes [35,50,79]. Thus, ATG9/ATG9A vesicles are thought to interact with the PAS transiently to provide lipids, transmembrane proteins, or vesicle-enclosed proteins necessary for autophagosome growth before returning to the cytosol (Figure 3; Model 1B) [50,80].

### ATG2/ATG2A-ATG9/ATG9A complex-mediated lipid transfer and equilibration (Figure 3; models 2A and 2B)

Recent in vitro studies conducted by three independent groups, however, have proposed a more direct involvement of ATG9/ATG9A in phagophore membrane expansion, acting as a lipid scramblase that equilibrates lipids across the bilayer (Figure 3; Models 2A, 2B and 3) [41,42,47]. In this model, a very small amount of ATG9/ATG9A on the phagophore membrane forms a protein complex with other proteins necessary for lipid transfer and equilibration [11,81]. Importantly, as we discussed earlier, a strong relationship exists between lipid scramblase activity and the structure of ATG9/ATG9A (see section 2 and Figure 2). According to the structural analyses, the central and lateral pores are sufficient to accommodate the phospholipids, and the internal cavity is rich in charged residues, allowing the hydrophilic heads of phospholipids to traverse the passage (Figure 2) [41,42]. Mutation analyses further revealed that mutations in the central or lateral pores significantly reduce the lipid scramblase activity of ATG9A, impairing the expansion of the phagophore membrane and autophagy; both pore mutations produce a significant defect, while central pore mutations lead to more severe phenotypes [41,42].

Thus, this model proposes that during membrane expansion of the growing phagophore, lipids need to be transferred from the ER (endoplasmic reticulum) and redistributed across a membrane bilayer [11,48,75,82–84] rather than being directly supplied by ATG9/ATG9A vesicles (Figure 3) [76,77]. This process is thought to occur through ATG2/ATG2A, a rod-shaped membrane tether protein, which transfers lipids from the ER to the growing autophagosome [47,48,75,82–84], whereas ATG9/ATG9A acts as a lipid scramblase that redistributes lipids across the bilayer [41,42,47]. The precise mechanism by which ATG2A is regulated has largely remained unknown. However, structural and biochemical analyses of this protein have suggested that it functions as a bulk lipid transporter, utilizing the large cavity inside [82,84]. Indeed, the location of ATG2/ATG2A at the ER-phagophore junction observed in yeast [83] and COS7 cells [82] supports this concept. In addition, a recent study documented the formation of a heterotetrametric complex by ATG9A and ATG2A [47,75]. Thus, ATG9/ATG9A is believed to translocate phospholipids that are delivered by ATG2/ATG2A for phagophore expansion from the cytoplasm to the lumenal leaflet (Figure 3; Models 2A, 2B and 3) [35,47,48,75].

However, other proteins are required for the proper function and localization of this complex. For example, the formation of the ATG2/ATG2A-ATG9/ATG9A complex at ER-phagophore contact sites necessitates the recruitment of ATG2/ATG2A to the phagophore, where ATG9/ATG9A is located. This recruitment process is believed to be mediated by WDR45/WIPI4 (Figure 3; Models 2A, 2B and 3), a well-known ATG2/ATG2A interactor that recognizes the lipid molecule PtdIns3P on the membrane close to ATG9/ATG9A and then recruits it to this location [11,85–87]. In addition, the ER-resident lipid scramblases TMEM41B (transmembrane protein 41B) and VMP1 (vacuole membrane protein 1) are known to interact with ATG2A [75]. Since lipid transfer from the ER to the phagophore likely generates lipid asymmetry in the ER [87], the function of these proteins is crucial for equilibrating the leaflets of the ER through their scramblase activity (Figure 3; Model 2A). Indeed, depletion of either protein results in autophagy defects [88–90]. Overall, WDR45/WIPI4 can aid in the proper localization of ATG2/ATG2A to the ER-phagophore contact site, and the protein complex formed by TMEM41B-VMP1-ATG2/ATG2A-ATG9/ATG9A subsequently acts as a lipid transport bridge, transporting lipids from the ER to the phagophore for phagophore expansion (Figure 3; Model 2A) [11,91]. This model is fascinating and fits well with many previous studies. However, a major mystery in this model has not yet been resolved. For phagophore growth, lipids need to be transported from a membrane source (in this case, the ER) to the phagophore. This transport implies a directional movement of lipids. Thus, an additional mechanism must be activated to ensure that lipids are transferred unidirectionally from the ER to the phagophore. One interesting idea (Figure 3; Model 2B) may explain how this transport occurs in cells. In this model, a lipid synthase on the ER synthesizes new lipids near the ATG2/ATG2A-ATG9/ATG9A complex (on the ER side), and the greater chemical potential of newly synthesized phospholipids might provide the driving force for the unidirectional transfer of lipids from the ER to the phagophore (Figure 3 Model 3; 2B) [48]. However, further details and precise molecular mechanisms need to be addressed in future research.

### Combined model (Figure 3; Model 3)

The two models described above propose different roles for ATG9/ATG9A in phagophore growth. However, recent studies suggest that ATG9/ATG9A may function at different stages of the autophagic process (Figure 3; Model 3) [35,92,93]. This model has been supported by recent observations [35]. In *ATG2A* and *ATG2B* double KO fibroblasts, ATG9A-positive vesicles cluster into a large cup-like morphology near the ER and colocalize with the initial autophagy proteins RB1CC1, WIPI2, and lipidated LC3B, but they fail to undergo the autophagosome membrane expansion stage [35]. These results suggest that ATG9A vesicles seed membranes for the initial stage of nucleation (Figure 3; Model 3), recruiting other autophagy initiation factors. Additionally, this process can occur independently of ATG2A/B interactions, but these interactions are required for phagophore expansion.

Therefore, the common feature of the two models (Models 2 and 3) is the role of the ATG2/ATG2A-ATG9/ATG9A complex in phagophore expansion and growth. This concept is intriguing but needs to be carefully examined in future studies. Many in vitro biochemical data support this idea [92,93], but limited information is available on whether this lipid transfer occurs in living cells. In addition, the colocalization of ATG2/ATG2A and ATG9/ATG9A in wild-type cells under a fluorescence microscope is not well defined. This result could be due to the low amount of ATG9A on the expanding phagophores [35,50], the transient localization of ATG9A on the autophagosomes [50,80], or the predominant localization of ATG2A on lipid droplets when it is overexpressed in cells [94,95]. Therefore, further research is essential to obtain a deeper understanding of this mechanism in cells. In particular, as we discussed above, addressing how the complex generates the driving force for certain lipids from the ER to the phagophore should be a priority in future research.

Additionally, the existence of the ATG2/ATG2A-ATG9/ATG9A complex in neurons is largely unknown. Upon careful examination of multiple independent proteomic studies investigating purified SV fractions [71,96,97] or immuno-isolated ATG9A vesicles [59] from nerve terminals, ATG2A was not found on any of the lists. This result might be due to the extremely low level of ATG2A in nerve terminals or its transient interactions with ATG9A. Alternatively, neurons may employ different membrane expansion mechanisms for presynaptic autophagy (Figure 4). Therefore, addressing the function of ATG9A in presynaptic autophagy is crucial for future research.

Indeed, recent research has shown potential interactions between ATG9A and other lipid transfer proteins [98]. For example, BLTP3B/SHIP164 (bridge-like lipid transfer protein family member 3B), which belongs to the bridge-like lipid transfer protein superfamily alongside ATG2A, exhibits structural and functional similarities to ATG2A and colocalizes with ATG9A upon overexpression in fibroblasts [98]. However, since overexpressed BLTP3B/SHIP164 does not colocalize with WIPI2 or LC3, this complex is unlikely to be involved in autophagy [98]. Another member of the bridge-like lipid transfer protein superfamily, VPS13A (vacuolar protein sorting 13 homolog A), is also known to interact with ATG9A but does not appear to participate in autophagy either [99]. Interestingly, loss-of-function mutations in VPS13A are known to cause chorea-acanthocytosis (ChAc), an autosomal recessive neurodegenerative disorder [100]. In neurons, VPS13A is predominantly expressed in the soma and neurites, where it colocalizes with markers of the ER and mitochondria, but it is not enriched in dendritic spines or synaptosomal fractions [101]. These findings further suggest that ATG9A-VPS13A interactions may occur in neurons to facilitate membrane expansion in other organelles, possibly in the soma and neurites rather than in synapses. However, the key interactors of ATG9/ATG9A in nerve terminals that mediate presynaptic autophagic processes remain to be identified.

### Organelle extrusion model (Figure 3; Model 4)

Some researchers have proposed that phagophores directly form at the ER subdomains known as omegasomes [77,102–104] or at the outer membrane of the mitochondria (Figure 3; Model 4) [105]. According to this hypothesis, the ER can directly [102] or indirectly, through the mitochondria via the ER-mitochondria contact site [105,106], supply membranes to the phagophore (Figure 3; Model 4). Therefore, ATG2/ATG2A-ATG9/ATG9A-mediated lipid transfer may not be necessary for this model. The precise role of ATG9/ATG9A is still unclear; however, transient and partial colocalization of ATG9A vesicles and tubular ER structures [79,102] or mitochondria [15] after starvation suggest a potential function of ATG9/ATG9A in the nucleation of autophagosomes on the ER or mitochondrial membranes (Figure 3; Model 4). In this case, since a lipid transfer process is not needed for phagophore growth, ATG9/ATG9A vesicles near the extrusion site may simply aid in the recruitment of other core components of the autophagy initiation machinery that bind to ATG9/ATG9A, such as the ATG13 and ULK1 complex [72,73], from the cytosol to this site to induce phagophore growth.

## Understanding the crucial roles of ATG9/ATG9A in deletion mutants

**Figure 5.**
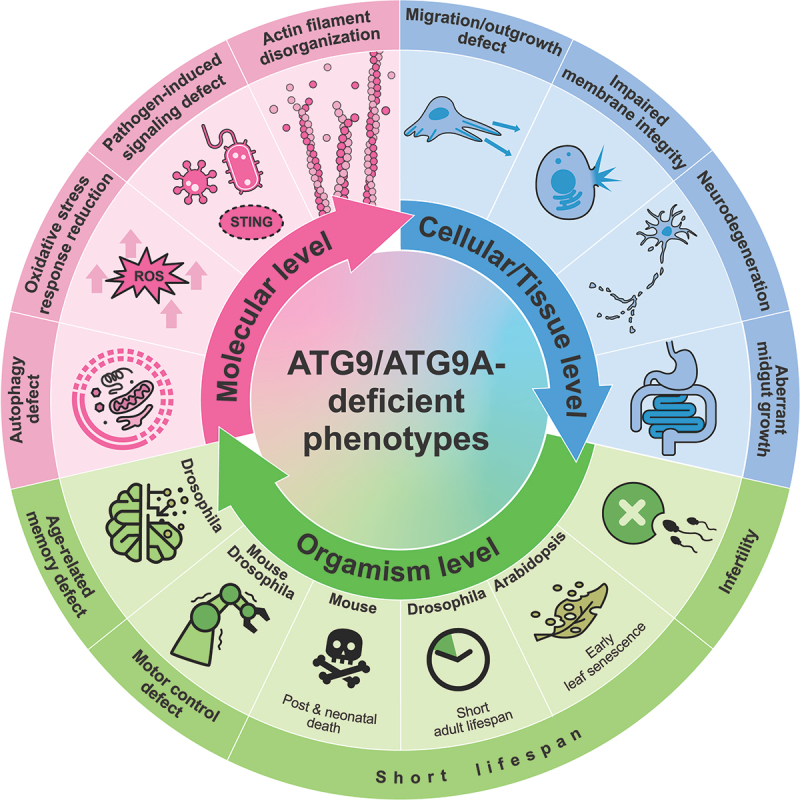
ATG9/ATG9A-deficient phenotypes. Loss of ATG9/ATG9A leads to various defects at different levels.

As previously described, ATG9/ATG9A is believed to have a main function in autophagy by interacting with numerous autophagy initiation factors and lipid transfer proteins, especially for the normal growth of autophagosomes [11–13,81]. However, ATG9/ATG9A also possesses various functions that do not directly involve autophagy. In this section, we explore the various functions of ATG9/ATG9A across species and discuss how these functions are linked to its deficient phenotypes (Figure 5).

At the organism level, ATG9/ATG9A proteins are essential for survival and longevity [29–36,107–110]. In *Drosophila*, the loss of *Atg9* leads to a shortened lifespan [107]. Similarly, in *Arabidopsis*, *ATG9* KO plants exhibit early leaf senescence [108], which can be considered premature aging since *Arabidopsis* undergoes its entire life cycle in 8 to 10 weeks [111]. In mice, the consequences of losing ATG9A are even more dramatic; these mice die during embryonic development or within one day after birth [29,30]. Interestingly, similar results were observed for animals with deficiencies in other autophagy-related proteins, such as ATG3, ATG5, ATG7, and ATG16L1, leading to embryonic death or postnatal death within one day [112–114]. Considering that a massive burst of autophagy occurs transiently soon after birth in normal neonatal mice [114], a deficiency in autophagy may lead to a critical shortage of amino acids, resulting in death within a short period. In worms, silencing *atg-9* significantly reduces the lifespan [109]; however, knocking down this protein after development tends to increase the lifespan [110]. The opposite developmental stage-dependent effects on lifespan were consistently observed in worms in which other autophagy-related genes were knocked down [110]. Taken together, the function of ATG9/ATG9A in regulating lifespan is likely related to its key role in autophagy, and autophagic dysfunction might lead to different effects on lifespan, depending on the developmental stage.

In addition, the sex-related function of Atg9 in reproduction has been suggested in *Drosophila* [115]. While *Atg9* KO males are fertile, KO females exhibit infertility, laying fewer eggs than wild-type females, and the laid eggs do not develop properly due to abnormalities in female gamete formation called oogenesis [115]. Since Atg9 is crucial for recruiting actin cable tip complex proteins, such as ena (enabled; the *Drosophila* homolog of VASP [vasodilator stimulated phosphoprotein]) and chic (chickadee; the homolog of PFN [profilin]), which are essential for the polymerization of the actin cytoskeleton, the absence of Atg9 results in improper nurse cell contraction, a process crucial for egg maturation, due to the disorganization of actin filaments [115].

Although researchers are still not certain whether ATG9A in mammals directly influences actin cable formation, some evidence has suggested its regulatory function in the organization of the actin cytoskeleton for numerous purposes, such as cell migration and neurite outgrowth, which are not directly related to autophagy [36,116]. For example, the depletion of ATG9A has been shown to alter the formation of lamellipodia, highly dynamic actin-based structures essential for directional migration, in human cell lines [116]. In addition, an increased interaction between ATG9A and IQGAPs, which are actin-binding scaffolding proteins, during plasma membrane damage, possibly for repair, has been reported [53]. In neurons, the loss of ATG9A results in impaired neurite extension [36], which is not observed in *Atg7* KO or *Atg16l1* KO neurons [117–120]. Moreover, brain-specific conditional KO (cKO) mice lacking ATG9A show impaired nerve fiber extension, potentially resulting in dysgenesis of the corpus callosum and anterior commissure [36]. Interestingly, similar phenotypes, such as a thin (but intact) corpus callosum and defective axons in various regions, have been observed in mice lacking the AP4E1 subunit [54]. In addition, as discussed earlier, dysfunction of AP-4 leads to massive accumulation of ATG9A in the TGN [31]. The abnormal accumulation of ATG9A at the TGN in *Ap-4 ε* KO neurons suggests a link between ATG9A dysfunction and hereditary spastic paraplegia/HSP (also referred to as AP-4 deficiency syndrome), a disorder characterized by progressive lower limb spasticity [56,57,121]. However, ATG9A trafficking defects and its massive accumulation at the TGN caused by *AP4E1/AP-4 ε* KO do not fully mimic the *atg9a* KO phenotypes [31,56]. Generally, *Atg9a* KO leads to more severe phenotypes, such as a drastically reduced lifespan and growth retardation [30]. These results may indicate that some portion of ATG9A can exit the TGN in the absence of AP-4 via an alternative pathway. Indeed, we previously showed that an AP-4 binding-defective mutant of ATG9A can exit the TGN but is abnormally colocalized with synaptophysin vesicle clusters when it is expressed together with synaptophysin in nonneuronal cells [46]. Additionally, an increase in total ATG9A expression in *AP4E1/AP-4 ε* KO cells may increase the chance of it being captured by other TGN exit pathways [31,56], possibly enabling these cells to recover from certain defects.

One obvious phenotype commonly observed in *Ap-4 ε* KO (whole KO) mice, *Atg9a* cKO (brain-specific KO) mice, and *Atg9* KO (whole) flies is locomotor defects [31,36,107]. Since locomotor impairment is a common phenotype of neurodegenerative disorders such as Parkinson disease [122], the function of ATG9A in neurons might be related to the progression of neurodegeneration. Indeed, abnormal accumulation of ATG9A in nerve terminals was observed in neurons from mutant mice carrying a synaptojanin1 missense mutation found in human patients with early-onset parkinsonism/EOP [123]. The SNCA/α-synuclein overexpression-induced mislocalization of ATG9A is also detected in fibroblasts [124]. These findings suggest the possible involvement of ATG9A in Parkinson disease. However, since locomotor defects are also found in flies with glial cell-specific knockdown of *Atg9* [125] and in animal models with cKO of other autophagy-related proteins [117–119], these defects might be due to the general autophagy defects that are crucial for maintaining cellular homeostasis and neural functions rather than the neural-specific functions of ATG9/ATG9A. Similarly, Atg9 deficiency in *Drosophila* causes age-related memory deficits, but the same phenotype is also observed in *Atg5* KO *Drosophila* [126].

Along with the functions mentioned above, various KO studies have further suggested multiple cellular roles of ATG9/ATG9A, including its involvement in the immune signaling pathway, plasma membrane protection [53,127], defense against pathogens such as influenza A virus [128] and HIV-1 [129,130], reduction of reactive oxygen species (ROS)-mediated stress [131], and maintenance of the integrity of the fly intestinal barrier (Figure 5) [107].

## Concluding remarks

In this review, we examined key research findings that shed light on the structure and function of ATG9/ATG9A in both nonneuronal and neuronal cells. As discussed, research on ATG9/ATG9A is still in its initial stages, with many characteristics remaining unknown, including its function, localization, biogenesis, trafficking, and interaction partners. Recent structural analyses have revealed detailed and unique pore structures, suggesting its potential role as a lipid scramblase. This finding has inspired the development of novel models of membrane expansion during autophagosome biogenesis. However, debates and inconsistencies between models exist, and very little is known about the function of ATG9/ATG9A in neuronal cells. Due to the small size and complexity of nerve terminals, investigating the role of ATG9/ATG9A in presynaptic nerve terminals is likely to be challenging. However, recent findings strongly suggest its distinct localization (not to SVs) in nerve terminals and its presumed independent interactome [46,59]. Proteomic data, especially from ATG9A vesicles isolated from nerve terminals [59], will provide us with insights into the role of ATG9/ATG9A in presynaptic autophagy and will guide our future investigations. One open question here is why only a small amount of ATG9A is detected in purified vesicles (3 copies per 100 vesicles) from synaptosomes [71] despite the high expression levels of ATG9A in the CNS [24]. Thus, ATG9/ATG9A in other parts of neurons or in other cell types in the brain, such as glial cells, may also play a crucial role in brain function. The high expression of Atg9 in glial cells [125] may support this idea. Finally, since *ATG9/ATG9A* KO in different species commonly leads to neurological defects found in neurodegenerative diseases [30,107,115], revealing the precise function of ATG9/ATG9A in cells will enhance our understanding of the underlying mechanisms of neurodegenerative diseases and underscore the importance of the autophagic process for normal neural functions. In addition, studying the function of ATG9/ATG9A in different types of cells and tissues will help us understand both its general and tissue-specific functions.
